# Assessment of Waist-to-Hip Ratio Attractiveness in Women: An Anthropometric Analysis of Digital Silhouettes

**DOI:** 10.1007/s10508-013-0166-1

**Published:** 2013-08-24

**Authors:** Krzysztof Kościński

**Affiliations:** Institute of Anthropology, Faculty of Biology, Adam Mickiewicz University, Umultowska 89, 61-614 Poznan, Poland

**Keywords:** Waist-to-hip ratio, Attractiveness, Preference, Mate choice

## Abstract

The low proportion of waist to hip size in females is a unique and adaptive human feature. In contemporary human populations, the waist-to-hip ratio (WHR) is negatively associated with women’s health, fecundity, and cognitive ability. It is, therefore, hypothesized that men will prefer women with low WHR. Although this prediction is supported by many studies, considerable disagreement persists about which WHR values are the most attractive and the importance of WHR for attractiveness of the female body. Unfortunately, the methods applied thus far are flawed in several ways. In the present study, we investigated male preferences for female WHR using a high precision assessment procedure and digitally manufactured, high quality, anthropometrically informed stimuli which were disentangled from body mass covariation. Forty men were requested to choose the most attractive silhouette consecutively from six series (2 levels of realism × 3 levels of body mass), each consisting of 26 female images that varied in WHR (from .60 to .85 by .01). Substantial inter-individual variation in the choices made was observed. Nevertheless, low and average WHR values were chosen more frequently than above-average values or values below the normal variation of the trait. This preference pattern mirrors the relationship between WHR and mate value, suggesting that the preferences are adaptive.

## Introduction

The low ratio of waist to hip size in females is a unique human feature (Singh, 1993) and several adaptive mechanisms might have contributed to its evolution. First, the human newborn has a relatively large head and a large pelvis facilitates its delivery (Rosenberg, 1992). Second, a narrow waist is a visual cue of the absence of pregnancy and therefore current fecundity—a feature that ancestral men sought in women. This may be an especially important cue in humans because women do not signal their present fertility in any other easily perceptible way (Singh, 1993). Third, fat, when deposited around the hips rather than the waist, facilitates bipedal stability of pregnant and lactating women (Pawłowski & Grabarczyk, 2003), contains fatty acids beneficial for brain development of the fetus and infant (Lassek & Gaulin, 2008), and may dishonestly signal a broad pelvis and absence of pregnancy so as to make the woman attractive to men (Furnham, Mistry, & McClelland, 2004; Low, Alexander, & Noonan, 1987).

In contemporary human populations, waist-to-hip ratio (WHR) is negatively related to the level of estradiol and positively related to the level of testosterone, which is why WHR is clearly lower in women than men (see literature cited in Singh & Singh, 2011). High WHR values in women are associated with mortality and many medical conditions, such as cardiovascular diseases, type 2 diabetes, gall bladder disease, lung function impairment, carcinomas, menstrual irregularity, anovulatory cycles, and subfertility (as reviewed in Singh & Singh, 2011; World Health Organization, 2011). According to sexual selection theory (Kokko, Brooks, McNamara, & Houston, 2002), these would have driven the evolution of male preference for low-WHR women which, in turn, would become another selective factor for low WHR. Although preference for low WHR was indeed found in most studies of industrial and traditional societies (reviewed in Singh & Singh, 2011), large inconsistency exists as to the most attractive value of WHR. Even if we limit our focus to populations of European descent, the values range from .5 (Heaney, 2000; Schützwohl, 2006), which is far below the normal range of the trait (see below) to .8, which is somewhat above the average (Henss, 1995), or even higher values (Furnham, Swami, & Shah, 2006). A more precise determination of the most attractive WHR would allow for the establishment of the type of sexual selection that acts on the body characteristic: whether the preference is for values close to the mean (.70–.75; stabilizing selection), below-average but within the range of normal variation (about .65; directional selection), or below the normal range (.60 or less; strongly directional selection). It would also facilitate confirmation of whether the preferred WHR values are those associated with good health (i.e., values somewhat below the average) or not (i.e., average, above-average or extremely low values) (Lassek & Gaulin, 2008; Riordan, 2007; Singh & Singh, 2011; Wass, Waldenström, Rössner, & Hellberg, 1997). Because previous research has not answered the question, further investigation is warranted.

The chief reason for inconsistency in results of previous studies seems to be methodological diversity and, in particular, the stimuli to be assessed. These include schematic drawings of females varying in WHR (e.g., Singh, 1993; Tassinary & Hansen, 1998), digitally manipulated photographs (e.g., Dixson, Grimshaw, Linklater, & Dixson, 2011; Rozmus-Wrzesińska & Pawłowski, 2005), non-manipulated photographs of real women (e.g., Thornhill & Grammer, 1999; Tovée, Maisey, Emery, & Cornelissen, 1999), movies with rotating three-dimensional figures (e.g., Fan, Liu, Wu, & Dai, 2004; Rilling, Kaufman, Smith, Patel, & Worthman, 2009), and pairs of photographs of the same woman before and after a surgical WHR reduction (e.g., Dixson, Li, & Dixson, 2010; Singh & Randall, 2007). In studies based on non-manipulated images of real women, estimation of the preferred WHR value is difficult because attractiveness assessments are confounded by body characteristics correlated with WHR, which in the first place is body mass (Rilling et al., 2009; Tassinary & Hansen, 1998; Tovée et al., 1999). Studies that statistically controlled for body mass (e.g., Brooks, Shelly, Fan, Zhai, & Chau, 2010; Cornelissen, Hancock, Kiviniemic, George, & Tovée, 2009; Cornelissen, Tovée, & Bateson, 2009; Fan et al., 2004; Rilling et al., 2009; Tovée et al., 1999; Tovée, Hancock, Mahmoodi, Singleton, & Cornelissen, 2002) usually reported that attractiveness was negatively correlated with WHR (with *r*s between 0.1 and 0.3), but the precision of such statements in identifying the most attractive WHR is unsatisfactory. Studies involving photographs of women before and after surgical WHR reduction can determine which of these two is the more attractive but does not reveal the most attractive possible value of WHR. Digital manipulation of silhouettes can potentially be the best approach, yet previous studies of this sort embodied several serious methodological weaknesses:

1. Many studies used schematic silhouette drawings developed by Singh (1993), which were extensively criticized for their poor realism and therefore unreliable results (Rilling et al., 2009; Tovée et al., 1999). The same criticism pertains to schematic drawings of the female body developed by other researchers (e.g., Marlowe, Apicella, & Reed, 2005; Tassinary & Hansen, 1998).
2. A change of waist width or hips width alone (whether applied to drawings or to high-quality photographs) influences not only WHR but also body mass and therefore body mass index (BMI); narrowing the waist or hips decreases BMI, while widening them increases BMI. If figures to be assessed differ from one another both in WHR and BMI, it is difficult to establish the most preferred WHR. Low-WHR silhouettes were usually obtained by narrowing the waist (rather than widening the hips) so the high ratings of silhouettes with marked waist incision obtained could reflect a preference for low BMI rather than low WHR (Rilling et al., 2009; Tovée et al., 1999). Importantly, low BMI is indeed preferred in industrialized populations (Brooks et al., 2010; Tovée et al., 1999).
3. The WHR of the prepared silhouettes were usually calculated as the ratio between *width* of waist and hips on two-dimensional images whereas the appropriate method is to determine the ratio between their *circumferences* (Rilling et al., 2009; Smith et al., 2007; World Health Organization, 2011). Estimations provided by both methods can differ to the extent that the shapes of horizontal section at the level of waist and hips differ from each other. In epidemiological studies, which showed that WHR is related to many health indices, the parameter was measured as the ratio of circumferences. Research on attractiveness should then use the circumference-based WHR to make their results comparable with epidemiological data and to establish whether the most preferable WHR coincides with that associated with the best health.
4. Raters viewed only a couple of female silhouettes, which usually had WHR values .7, .8, .9, and 1.0. This decreases the precision of estimation of the most attractive WHR.

In the present study, we attempted to determine the most preferred female WHR by using silhouette stimuli free of the abovementioned weaknesses. First, stimulus images were made on the basis of a photograph of a woman possessing body proportions typical for local (Polish) females. Second, relying on anthropometric data for the three-dimensional shape of the female body, rear-view silhouettes of various WHR but equivalent BMI were manufactured (rear view imaging was used to exclude the confounding effects of face and breast appearance on attractiveness perception). Third, for each two-dimensional female silhouette, circumference-based WHR was estimated. Finally, WHR of women depicted by the silhouettes ranged from .60 to .85 by .01, which allowed participants to more precisely express their preferences.

## Method

### Participants

Participants were 22 young white Polish women (aged 18–28 years) and 40 men (aged 18–39 years). The women were recruited opportunistically among students (*n* = 9) and sunbathers (*n* = 13) in Poznań, a relatively large city in western Poland. All were of apparent normal physique and were photographed and measured in order to prepare visual stimuli for attractiveness assessments. The men were recruited opportunistically among students (*n* = 29) and school teachers (*n* = 11) in Poznań. They were asked to assess attractiveness of the manufactured female silhouettes. The project of this study was approved by the Institutional Review Board at Poznan University of Medical Sciences (726/11).

### Measures

#### Construction of the Initial Silhouette

The female participants were photographed from the back and the right profile at a distance of 3 m with a digital camera (Panasonic DMC-FZ18, 8.1 MPx). They were clothed in only their underwear or a bikini. The women reported their height and weight, and their waist and hip circumferences were measured. Waist circumference was taken at the level of the smallest body width between ribs and hips and hip circumference at the level of the maximum body width below the waist. Although several methods of measuring waist and hip circumference exist (Cashdan, 2008; World Health Organization, 2011), the one applied here allows a direct comparison between the obtained WHR and those based on waist and hip widths obtained from photographs (as mentioned above, the latter procedure has frequently been applied in attractiveness research). Neither WHR (range = .65–.81, *M* = .75, *SD* = .05) nor BMI (range = 17.5–24.9, *M* = 20.6, *SD* = 2.3) was related to the women’s age (*r* = .26 and −.03).

Six of the women with apparently typical physique were measured for a further set of anthropometric traits and compared with average values for Polish women (for details, see Kościński, 2012). In this way, the woman possessing body proportions closest to the population means was identified. She was 22 years old, 167.5 cm tall, 53.5 kg in mass, and had a BMI of 19.1 and a WHR of .70. Although she had somewhat lower body mass and WHR than the population averages (58.7 kg and .72), these parameters were manipulated in her photograph during the stage of production of the final stimuli. In the rear-view photograph of this woman, the left body side was reflected and superimposed on the right to achieve an ideally symmetric figure. The legs were digitally lengthened by an equivalent of 1.7 cm at the expense of trunk length so as to make the relative leg length equal to the population average (this was accomplished by the method of warping; see below for details). The shoulder width, chest width, and upper limb length of the woman required no alteration.

#### Building of a Model of the Female Body

**Fig. 1:**
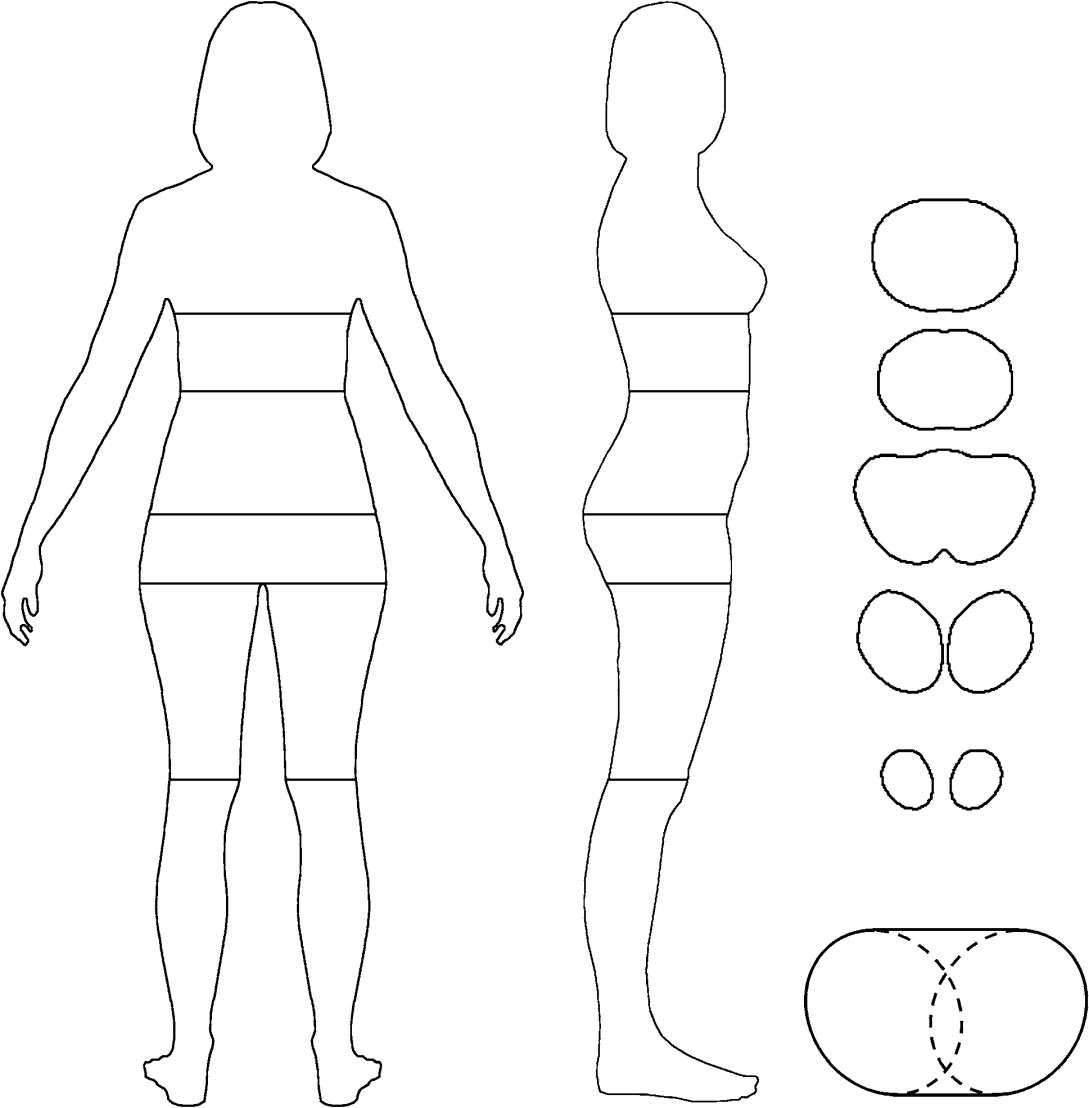
Analysis of female body shape. *Left* to determine depth-to-width proportions, rear- and profile-view horizontal dimensions of 22 real women’s silhouettes were measured at 100 levels, including 20, 30, 10, and 40 levels in breast-waist, waist-buttocks, buttocks-crotch, and crotch-knees segments. *Right* shapes of horizontal body sections at chest, waist, buttocks, crotch, and knees were obtained from an analysis of Victoria, a three-dimensional model of a woman’s figure (body fronts are upwards). *Right bottom* estimation of hip circumference with two half-ellipses and two line segments. Two ellipses, with one axis equal to hip depth and the second equal to half of hip width, were enlarged by 4 %, rotated to fit the lateral body outline and cut at their topmost and bottommost points. The total length of line segments in front and back of the body was 90 % of the hip width. The Peano’s formula for ellipse perimeter was applied: *π* × [1.5 × (width/2 + depth/2) − √(width/2 × depth/2)]

The silhouette representing the average body proportions for Polish women was further modified to produce figures of differing BMI and WHR values. Although a change in BMI that does not impact on WHR can be easily achieved by an appropriate change in overall body width, the modification of a rear-view female silhouette for WHR without producing a change in the BMI of the depicted woman is more challenging and requires a simultaneous change in both waist and hip width. Because we did not manipulate the silhouettes’ height, the invariance of BMI requires invariance in body mass, which can be approximated by body volume. The influence of waist and hip manipulations was restricted to the breast-knee part of the body (Fig. 1); further analysis was therefore confined to this region only.

We modeled the breast-knee region of the female body as an orderly vertical pile of 99 cylinders with irregular bases as obtained by horizontal sections of this region at 100 levels (Fig. 1), and determined how the size and shape of this region in a rear-view silhouette translates to the volume (and weight) of this body part. To this end, ratios between anteroposterior and transverse body dimensions (depth-to-width ratios) at 100 levels between breasts and knees for all 22 photographed women were calculated by dividing body widths (in pixels) on the profile-view image by the corresponding body widths on the rear-view image (Fig. 1). These depth-to-width ratios were positively correlated with BMI for levels from the upper part of thighs upwards: *r* = .44 (df = 20, *p* = .04) for the level of narrowest waist, *r* = .57 (df = 20, *p* = .006) for the most protruding buttocks, and *r* = .35 (df = 20, *p* = .11) at the crotch level. For middle and lower thighs, however, the relationship tended to be negative: *r* = −.30 (df = 20, *p* = .17) at the mid-thigh, and *r* = −.35 (df = 20, *p* = .11) at the knee level. Regression analyses of the depth-to-width ratio on BMI were then performed for a total of 100 levels between the breasts and knees and the results used to estimate depth-to-width ratios for the subsequently constructed silhouettes of varying BMI values.

To determine the shape of the base of the cylinders constituting the model of the body, horizontal body sections were produced with Bryce 7 software on a popular three-dimensional digital model of an anatomically correct woman named Victoria (www.daz3d.com). Because Victoria’s BMI is below-average (as can be easily assessed with the naked eye) and depth-to-width ratios depended on BMI, we used the model to determine the shapes of horizontal body sections only, but not depth-to-width ratios. The body sections were made at five levels: just below breasts, at the level of narrowest waist, greatest protrusion of the buttocks, crotch, and knees; with the superior views of the sections being saved to JPG files (Fig. 1). The ratio between the area of each body section and the area of the rectangle circumscribed around this section (i.e., the percentage of body pixels in this rectangle) was then determined. Ratios for the remaining body levels were obtained by linear interpolation.

The above-mentioned data enabled estimation of the anteroposterior dimensions and body volume between the breasts and knees for any rear-view silhouette. The total volume was the sum of the volumes of 99 cylinders with irregular bases. The volume of each cylinder was the product of its height and the area of its base. The height of the cylinder was taken as the height of the body part embracing the cylinder (e.g., hip-knee segment) divided by the number of cylinders in the body part (Fig. 1). The area of the base was the product of three parameters: body width, body depth, and the percentage of body area on the section at the appropriate level (more precisely, the parameters for a cylinder’s base were averages of these taken for two adjacent levels).

#### Production of Stimuli Silhouettes

**Fig. 2:**
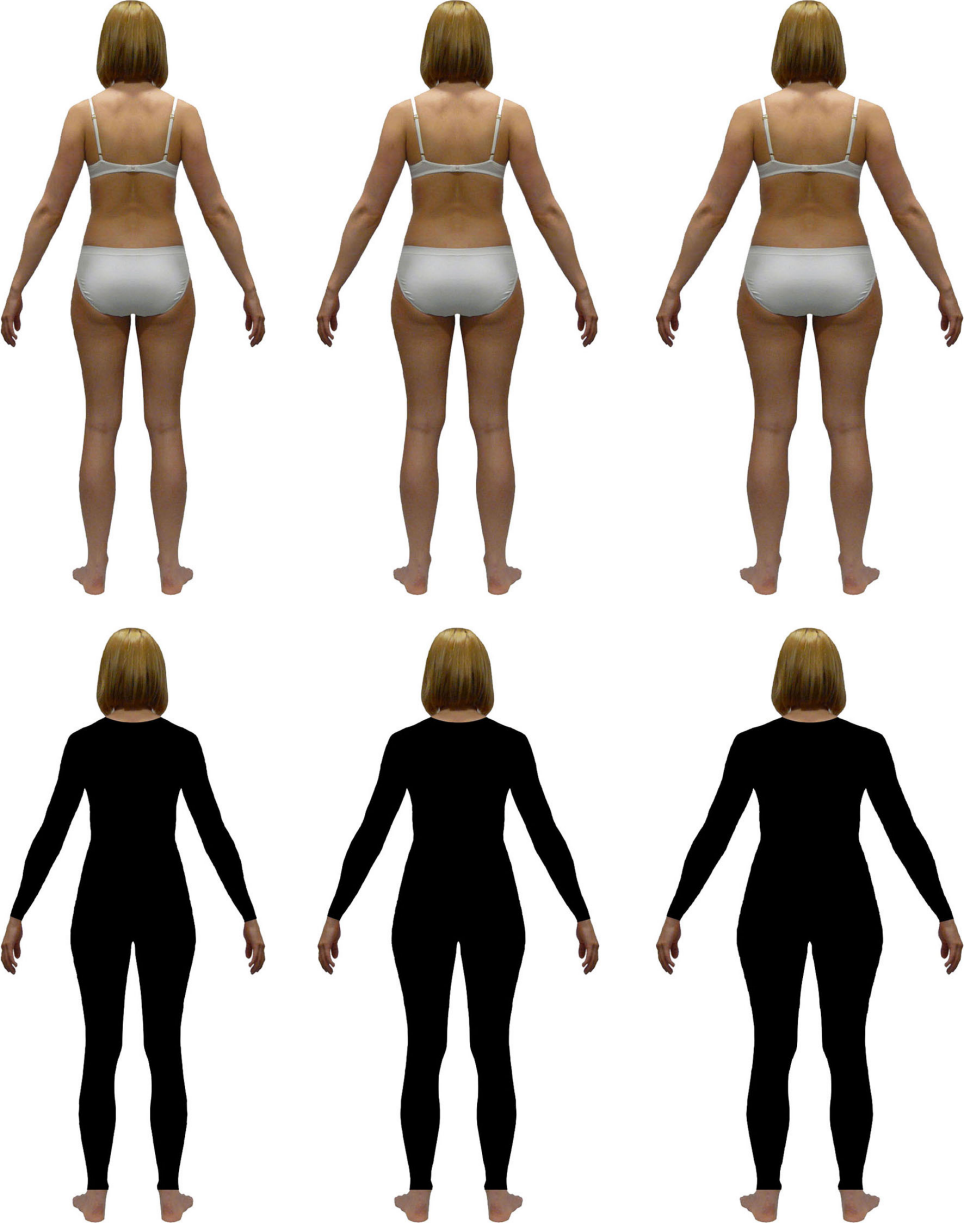
Color (*top*) and black (*bottom*) versions of silhouettes representing women with typical body proportions but diversified in body mass: light (*left*), average (*middle*), and heavy (*right*), corresponding to body mass index of 18.5, 21, and 25. Each silhouette was further digitally manipulated to produce figures varying in WHR (Color figure online)

All calculations of digital manipulations to be applied to the silhouette images were performed in the Microsoft Excel application. First, we determined the percentage changes in overall body width (excluding head) to be applied to the initial silhouette (BMI = 19.1) in order to produce silhouettes representing women with BMI of 21.0 (the average for young Polish women) and 18.5 and 25.0, which are the lower and upper limits of the medically normal range for BMI (World Health Organization, 2012). We assumed that a change of *X* percent in body width corresponds, in real individuals, with the same *X* percent change in body depth, which results in body weight being (1 + *X*/100)^2^ of the original weight.^1^ The width of the initial silhouette was then changed by −1.6, +4.8, and +14.4 % to obtain figures representing a light (BMI = 18.5), average (BMI = 21.0), and heavy (BMI = 25.0) woman, respectively. Besides these highly realistic silhouettes, an additional version of each was prepared in which whole bodies, excepting the head, hands, and feet, were blacked out as if the woman was wearing a leotard (Fig. 2).

Each of the thus produced six silhouettes (3 BMI levels × 2 color versions) was further modified in WHR value. The waist circumference of the woman depicted in a rear-view silhouette was calculated as the perimeter of the ellipse determined by the width and depth of the woman’s body at the level of narrowest waist (Fig. 1). Body shape of the section at widest hips was not elliptic, and, therefore, the hip circumference was estimated with a carefully constructed combination of elliptic and line segments (Fig. 1). The ratio of the thus assessed waist and hip circumference provided the WHR estimate for the silhouette. To test the accuracy of the method, we compared WHR estimated by this method with WHR based on waist and hip circumferences measured on 22 live women. The estimated values were on average larger than the measured ones by .011 and correlated with them at .90. The disparities might have resulted from an imprecision of the algorithm applied and/or anthropometric measurement error. In any event, the estimated WHR corresponded very closely to the measured one. WHR values determined with each method were further compared with the ratio of waist width to hip width on the rear-view photographs. Although the width-based WHR was highly correlated with the circumference-based measured (*r* = .79) and estimated (*r* = .91) WHR, it was systematically lower than these, on average, by .037 and .048, respectively. This means that the ratio of waist width to hip width substantially underestimates the true WHR.

The Solver add-in for the Microsoft Excel application was used to determine the magnitude of waist and hip width changes required to obtain a silhouette depicting a woman who possessed the desired WHR and whose BMI was equal to the BMI of the original silhouette. This was facilitated by spreadsheet formulas that calculated anteroposterior body dimensions, the volume of the breast-knee segment, and waist and hip circumference on the basis of data on body widths of a rear-view female silhouette. For each BMI level (18.5, 21.0, and 25.0), the Solver was run 26 times so as to obtain silhouettes possessing WHR from .60 to .85 by .01. This range covered the normal WHR variability in young Polish women, which is about .64–.85 (Jasieńska, Ziomkiewicz, Ellison, Lipson, & Thune, 2004; Pawłowski & Grabarczyk, 2003), and also included some lower values so as to test whether the most attractive WHR falls below the normal range as was suggested in some studies (Heaney, 2000; Schützwohl, 2006).

**Fig. 3:**
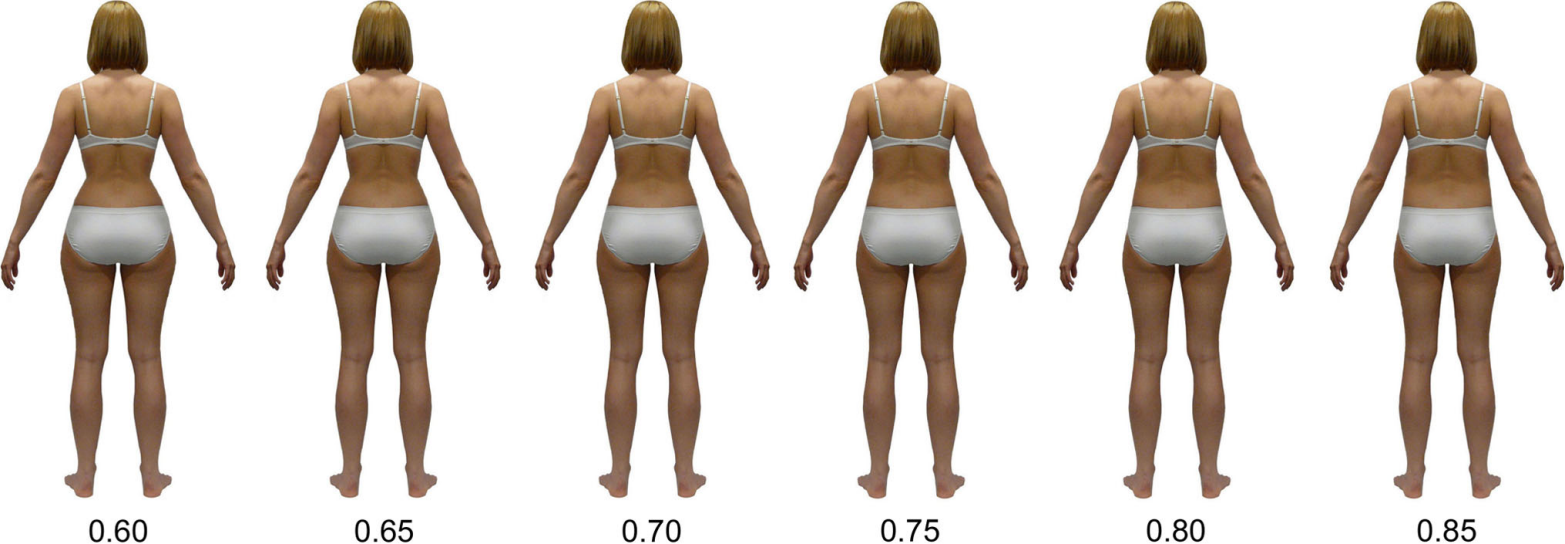
Examples of stimuli silhouettes: color version of average-weight woman (body mass index equal to 21) with WHR being .60, .65, .70, .75, .80, and .85. Altogether, stimuli included six series (color/black × light/average/heavy), each containing 26 silhouettes varying in WHR from .60 to .85 by .01 (Color figure online)

The magnitude of changes in overall body width (to alter BMI) and in waist and hip width (to alter WHR) as determined in Microsoft Excel was then graphically applied to images of rear-view silhouettes using author-developed software (in Microsoft Visual Basic 6). Images were manipulated by means of warping, a common technique for image distortion used in many studies on attractiveness of faces (e.g., Perrett, May, & Yoshikawa, 1994) and silhouettes (e.g., Kościński, 2012). The resultant 156 images (2 color versions × 3 BMI levels × 26 WHR levels) were saved to JPG files (Fig. 3).

### Procedure

The male participants were each given a web page address and asked to complete the questionnaire therein. The questionnaire interface was developed in Macromedia Flash MX 6. First, they provided their age, height, body mass, and sexual orientation (the question “What is your sexual orientation?” was answered using the Likert scale ranging from 1 = definitely homosexual, to 5 = definitely heterosexual; answer refusal permitted). Then, a female silhouette was displayed on the left-hand side of the screen with a resolution of 320 × 637 pixels and on the right-hand side appeared a vertical scrollbar and an instruction to use the scrollbar so as to make the silhouette as attractive as possible. The bar of the scrollbar could be set with the computer mouse at any of 26 locations corresponding to the 26 silhouettes in the loaded series. The image depicting the silhouette was dynamically replaced with another as the participant moved the bar. When the task was done, the participant clicked the “OK” button. Then the screen turned white for half a second and a silhouette from another series appeared along with the scrollbar. Because there were 6 silhouette series (2 colors × 3 BMI), the task of choosing the most attractive female silhouette was performed 6 times, providing values for 6 variables: Black18, Black21, Black25, Color18, Color21, and Color25. The order of series in respect of color (3 black series followed by 3 color or vice versa) and BMI (18.5–21–25 or 25–21–18.5), as well as WHR of the initially displayed figure (.60 or .85), was varied randomly between participants.

## Results

The male participants were aged 18–39 years (*M* = 26.1), 166–193 cm tall (*M* = 178.9), and weighed 55–100 kg (*M* = 77.0). Each reported to be definitely heterosexual, but one declined to answer and another described himself as bisexual. Because no man reported as being homosexual, all the participants were retained for further analysis. A GLM analysis with BMI (low/average/high) and coloration (black/color) as repeated variables, and age, height, and weight as covariates did not reveal any main or interaction effect of age, height or weight for WHR preferences (all *p*s > .10), and the variables were therefore not further analyzed. The assumption of sphericity was not violated (*χ* ^2^ = 1.30 and 2.65 for BMI and BMI × coloration, respectively), thereby validating the repeated measures analysis.

Mean WHR values perceived by men as the most attractive in six series of female silhouettes were about .7 (Table 1). Because the middle value of the WHR scale (.60–.85) was .725, and the average WHR for young Polish women is equal to or only slightly higher than this (Jasieńska et al., 2004; Pawłowski & Grabarczyk, 2003; Pokrywka, Čabrić, & Krakowiak, 2006), we tested whether the most preferred values differed significantly from .725. According to the one-sample *t* test, only values for Color18, *t*(39) = 3.12, *p* = .003, and Color21 series, *t*(39) = 2.53, *p* = .015, differed significantly from and was lower than .725.

**Table 1:** WHR values preferred by 40 men in six series of female silhouettes

Series	*M* (*SE*)	Frequencies of WHR intervals					*p*	*p*′
		.60–.64	.65–.69	.70–.74	.75–.79	.80–.85		
Black18	.71 (.011)	4	16	8	6	6	.016	.005
Black21	.72 (.010)	3	11	13	9	4	.025	.005
Black25	.73 (.012)	6	6	10	9	9	ns	ns
Color18	.70 (.008)	5	13	13	8	1	.004	.001
Color21	.69 (.010)	8	12	11	6	3	.077	.013
Color25	.72 (.010)	3	8	17	6	6	.004	.002

*p*s and *p*′s stand for *p*-levels in the Pearson’s *χ* ^2^ goodness-of-fit test. *p*s are for hypothesis of discrete uniform distribution of male choices (df = 4). *p*′s are for hypothesis of proportional frequencies for 2nd and 3rd intervals versus the other three intervals (df = 1). Calculations considered that the last range is wider than the others (6- vs. 5-category)

We then conducted a GLM analysis with BMI and coloration as repeated variables and order of series in respect of color and BMI, and the initial WHR value as grouping variables. The analysis revealed the main effects of silhouette coloration, *F*(1, 36) = 8.78, *η* ^2^ = .20, *p* = .005, BMI, *F*(2, 72) = 5.93, *η* ^2^ = .14, *p* = .004, and the order of series in respect of color, *F*(1, 36) = 4.37, *η* ^2^ = .11, *p* = .044; no interaction term was significant. These effects indicated that a lower WHR was preferred on color (*M* = .71) than black silhouettes (*M* = .72), on light (*M* = .71) and average (*M* = .71) than heavy silhouettes (*M* = .73), and when black (*M* = .70) rather than color (*M* = .73) silhouettes were presented first in the series.

Besides mean values, Table 1 provides the frequencies of choice for five WHR intervals. Although inter-individual variation in the most preferred WHR was large, pairwise correlations between WHR values chosen in different series were .38–.68 and Cronbach’s alpha for all six series was .88, indicating fairly good intra-individual consistency. Furthermore, there was a tendency for .65–.69 and .70–.74 intervals to be chosen more frequently than the three other. The Pearson’s *χ* ^2^ goodness-of-fit test rejected a hypothesis of the discrete uniform distribution for 4 of 6 series (see *p*-levels in Table 1) and a hypothesis of proportional frequencies for .65–.69 and .70–.74 intervals versus the three other intervals for 5 of 6 series (see *p*-levels in Table 1). These results indicate that men usually preferred low-to-average WHR values to above-average values or values below the normal variation of the trait, and this was especially true when they assessed color (i.e., more realistic) silhouettes or those of low or average BMI.

## Discussion

The present study investigated male preferences for female WHR using a high precision assessment procedure and digitally manufactured, high quality, anthropometrically informed stimuli which were disentangled from BMI covariation. The preference showed a large inter-individual variance; nevertheless, low-to-average WHR values (i.e., .65–.75) were usually regarded the most attractive. Above-average values (>.75) or values below the normal variation of the trait (<.65) were preferred less frequently. Very low WHR makes a woman’s appearance atypical and this may decrease her attractiveness (Kościński, 2012). Deviations from normal appearance frequently signal poor health (see references in Kościński, 2012) and this may pertain to WHR as well. Indeed, very narrow waist limits the space for abdomen organs and muscles, thus impairing their function (Riordan, 2007), while very broad hips are costly to develop and impair locomotion (Low et al., 1987). Furthermore, somatic health (Singh & Singh, 2011) and fecundity (Wass et al., 1997) are relatively low when a woman’s WHR is above-average but are not associated with WHR within its low-to-average range. Fat deposited around the hips contains fatty acids beneficial for brain development and the women’s cognitive ability was found to be negatively related to their WHR; again, this relationship occurred within the average-to-high rather than the low-to-average range (Lassek & Gaulin, 2008). Therefore, the presently observed pattern of male preferences for female WHR mirrors the relationship between WHR and mate value, suggesting that the preferences are adaptive. These claims might be further tested on other populations, in particular those that possess WHR higher than Poles, which is the case for most populations (Cashdan, 2008; Marlowe et al., 2005; Molarius, Seidell, Sans, Tuomilehto, & Kuulasmaa, 1999).

Present results may reconcile two contesting positions on whether WHR plays major (Singh & Singh, 2011) or little (Tovée et al., 1999) role for women’s attractiveness. In agreement with Singh’s view (Singh & Singh, 2011), we found that the statistically most preferred WHR value was very close to .70, given that men assessed a realistic (color) figure of a non-overweight woman. In accordance with Tovée’s view (Tovée et al., 1999), despite the average of .70, the most preferred WHR varied enormously among male judges and there was only a moderate tendency to choose low or average rather than high WHR as the most attractive. Inter-study disagreement on the role of WHR in female attractiveness and the relative importance of WHR and BMI arguably results from methodological variation and weaknesses, including the use of schematic silhouette drawings of a poor realism or of photographs of real women without having controlled for all of the confounders. The importance of WHR may be investigated in future research with stimuli similar to those presently used in a more direct way (e.g., raters may evaluate attractiveness of individually seen silhouettes).

We also found that lower WHR values were preferred in color (i.e., more realistic) than black silhouettes, in light and average than heavy silhouettes, and when black rather than color silhouettes were presented first. A stronger preference for low WHR in women having low or average (as opposed to high) body mass has already been observed in previous research (Davis & Cerullo, 1996; Singh, 1993). The relation of attractiveness impairment from wide hips to attractiveness enhancement from a small waist seems therefore to increase with a woman’s body mass. The effect of stimulus realism on preferences strengthens doubt about the accuracy of results obtained in studies involving line drawings of the female body (see Introduction) as well as in studies that used gray-scale photographs in which the head and/or limbs were not visible (e.g., Smith et al., 2007; Tovée et al., 2002). The reason for the observed effect of the silhouettes’ presentation order on preference for WHR is not clear.

Finally, we demonstrated that the calculation of WHR as the ratio of waist and hip widths, as has frequently been done in attractiveness research, noticeably underestimates the true WHR which is the ratio of girths measured at the respective levels. This difference amounts to .04, which is about 1 SD (Pawłowski & Grabarczyk, 2003; Pokrywka et al., 2006) and means, for example, that a woman with WHR of .70 would possess the width-based WHR of about .66. The factor of 1.055 provided by Smith et al. (2007) leads to very similar conclusions (.66 × 1.055 = .70). This bias should be borne in mind when WHR values which are calculated from measurements obtained from front- or rear-view photographs are compared with measurements from live subjects.

### Conclusions

The main conclusions from the present study are as follows:

1. Although men tend to prefer women having low WHR, the inter-individual variance in preferences is large and lower WHR is preferred in light and average than heavy women. This indicates that the preference for WHR, and possibly human physical preferences in general, cannot be encapsulated in a simple statement, such as that the most attractive WHR value is .7.
2. Attractiveness and health are related to WHR in a similar way (each being highest for its low and average values), thereby supporting the evolutionary psychological view that male preference for female body shape is adaptive.
3. Lower WHR values were preferred for color (i.e., more realistic) than black silhouettes, and the width-based estimation of WHR obtained from front- or rear-view photographs noticeably underestimates the true, circumference-based WHR obtained from measurements of live subjects. These findings compound the doubt about the accuracy of results obtained in previous studies and underscores the importance of stimulus quality in research on human preferences.
4. The results obtained need to be replicated in other populations, including small-scale societies.

## Footnotes

1 Note that Wilson, Tripp, and Boland (2005) incorrectly changed the width of the silhouette by *X* percent to alter its BMI by *X* percent.
